# Editorial: Advances and Challenges in Nanomedicine

**DOI:** 10.3389/fphar.2018.01397

**Published:** 2018-11-29

**Authors:** Susan Hua, Sherry Y. Wu

**Affiliations:** ^1^Therapeutic Targeting Research Group, School of Biomedical Sciences and Pharmacy, University of Newcastle, Callaghan, NSW, Australia; ^2^Hunter Medical Research Institute, New Lambton Heights, NSW, Australia; ^3^School of Biomedical Sciences, University of Queensland, Brisbane, QLD, Australia

**Keywords:** nanomedicine, nanotechnology, nanoparticles, targeted drug delivery, translation

The use of nanotechnology in medicine has the potential to have a significant impact on human health by improving the diagnosis, prevention and treatment of diseases. Nanomedicines typically encapsulate therapeutic and/or imaging compounds in submicrometer-sized carrier materials. In the last several decades, the application of nanomedicine for clinical purposes has received significant attention from academia, researchers, government, funding agencies, and regulatory bodies (Allen and Cullis, 2004; Sercombe et al., 2015; Hare et al., 2017; Hua et al.). Nanomedicines are generally intended to increase the therapeutic index of compounds by allowing more efficient delivery to the target site to enhance therapeutic efficacy and/or by minimizing accumulation in healthy body sites to reduce toxicity. Nanoencapsulation can also protect therapeutics from degradation in biological environments and can provide solubilization (Talekar et al., 2015; Mishra et al., 2017; Shajari et al., 2017). This e-Book focuses on articles that discuss the advances and challenges in the nanomedicine field across a broad range of topics. A brief summary of each article is provided below.

Nanomedicines can have a combination of chemical, physical, and biological properties that influences their *in vivo* behavior.

- Arms et al. addressed the principles and methodology of the available techniques for evaluating *in vivo* biodistribution of nanoparticles (Arms et al.). They further compared the advantages, limitations and capabilities of the techniques for assessing cellular, whole-organ and real-time accumulation (Arms et al.). Determining the *in vivo* biodistribution of nanoparticles following administration in animals and humans is an important component in the translational assessment of nanomedicines (Arms et al.).
- In addition, Robson et al. discussed the advantages and limitations of available imaging techniques used to evaluate the morphology of liposomal formulations (Robson et al.). Validating and controlling the morphology of nanoparticles is important for clinical translation, however it is generally difficult to control and not well-characterized (Robson et al.).

There are a number of factors that can impose significant obstacles to the clinical translation of nanomedicines, irrespective of whether they are therapeutically beneficial or not.

- Accomasso et al. summarized the current state regarding the safety evaluation of nano-based therapeutics and discussed the importance of risk assessment and risk minimization in the development of nanomedicines (Accomasso et al.). With the rapid growth in the use of nanomaterials for medical applications, the most urgent need is developing and validating novel and practical approaches that are able to determine potential short-term and long-term health risks, including the extrapolation of acute *in vitro* results for the prediction of chronic *in vivo* effects (Accomasso et al.).
- More broadly, Hua et al. discussed the current trends and challenges in the clinical translation of nanoparticulate nanomedicines, as well as the potential pathways for translational development and commercialization (Hua et al.). Key issues related to the clinical development of nanoparticulate nanomedicines include biological challenges, biocompatibility and safety, large scale manufacturing, government regulations, intellectual property (IP), and overall cost-effectiveness in comparison to current therapies (Hua et al.).

Undoubtedly, the vast majority of nanomedicines in preclinical and clinical development as well as in clinical use are for targeting a wide variety of malignant tumors.

- Yang et al. reviewed the advances and challenges in utilizing exosomes for the delivery of cancer therapeutics (Yang and Wu). Significant progress has been made to overcome major barriers for using exosomes as a delivery system, thereby opening a new promising avenue for cancer treatment (Yang and Wu).
- Chang et al. highlighted the current understanding and challenges of biologically targeted magnetic hyperthermia to induce cancer cell death and potentially improve the effectiveness and safety of hyperthermia (Chang et al.). Application of an alternating magnetic field following administration of cancer-targeting magnetic nanoparticles that accumulate in the tumor allows preferential heating of malignant cancer cells (Chang et al.). Despite promising results in preclinical studies, there are a number of challenges that still need to be addressed before this technique can progress to the clinic (Chang et al.).
- Furthermore, Buss et al. discussed how nanotechnology can help overcome current obstacles for the treatment of bladder cancer. This includes how it can be used in non-muscle-invasive urothelial bladder cancer to facilitate combination chemotherapeutic and BCG (mycobacterium bovis bacillus Calmette–Guerin) immunotherapies (Buss et al.).

Modulation of the tumor microenvironment has recently emerged as an important strategy to improve the delivery of nanomedicines to tumors, given the importance of cancer-associated cells in tumor growth and metastasis.

- Zhang et al. discussed the existing approaches and strategies for modulating the tumor microenvironment to improve tumor perfusion (Zhang et al.). This enables accumulation of nanomedicines at the tumor site, facilitates extravasation of nanomedicines for improving transvascular transport, and enhances interstitial transport for optimizing the biodistribution of nanomedicines (Zhang et al.). These strategies may provide an opportunity for the development of novel combination chemotherapeutic regimens and reassessment of previously suboptimal compounds (Zhang et al.).
- Correspondingly, Harrison et al. addressed the advances and challenges of nucleic acid delivery of nanoparticles to the tumor microenvironment (Harrison et al.). Despite the development of various nanoparticle platforms to overcome nucleic acid delivery hurdles, several challenges still exist for effective tumor delivery (Harrison et al.). One such challenge has been the accumulation of nanoparticles in non-cancer cells within the tumor microenvironment, which has recently opened up novel therapeutic applications for nanoparticles (Harrison et al.).

The application of nanomedicine-based therapies for drug targeting to non-cancer conditions has also increased in recent years.

- Remiao et al. presented recent developments in nanotechnology to overcome impairments still faced by medically assisted reproductive technology (e.g. multiple pregnancy, ovary stimulation, and genetic disorders) and new perspectives for the further use of nanotechnology in reproductive medicine (Remião et al.). The application of nanotechnology approaches to reproductive medicine have provided strategies to improve diagnosis and increase specificity and sensitivity (Remião et al.).
- Fang et al. showed that liposome-encapsulated baicalein may have the potential to improve wound healing and restore skin structure after skin injury (Fang et al.). The study demonstrated that liposome-encapsulated baicalein can inhibit adipogenic differentiation medium (ADM)-induced lipid accumulation and extracellular matrix formation in Hs68 fibroblasts through the suppression of lipogenesis enzymes and inflammatory responses (Fang et al.).
- In addition, Yan et al. have elucidated the possible pathways for layered double hydroxide (LDH) nanoparticles to enhance antigen (cross)-presentation on immune cells as adjuvants for protein vaccines (Yan et al.). Nanoparticles have been intensively investigated as adjuvants in new generation vaccines, however how these nanoparticles provoke immune responses is not well-understood (Yan et al.). This research would help to understand the nanoparticle adjuvant mechanism and further assist the design of new specific nanoparticles as more efficient nano-adjuvants (Yan et al.).

## Summary

Overall, nanomedicine has the potential to revolutionize the way we detect and treat damage to the human body. Although nanomedicines have demonstrated significant therapeutic advantages for a multitude of medical applications, their translation has not progressed as rapidly as the plethora of positive preclinical results would have suggested (Luxenhofer et al., 2014; Sercombe et al., 2015; Hare et al., 2017; Hua et al.). The experimental development of nanomedicines is continually progressing at a fast pace, however significant challenges still exist in promoting these platforms into clinically feasible therapies. Therefore, continued translational success will require communication and collaboration between experts involved in all stages of pharmaceutical development of nanotechnologies, including pharmaceutical design and manufacturing, cellular interactions and toxicology, as well as preclinical and clinical evaluation (Hua et al.).

## Author contributions

SH drafted the manuscript. SH and SW critically reviewed the manuscript for important intellectual content.

### Conflict of interest statement

The authors declare that the research was conducted in the absence of any commercial or financial relationships that could be construed as a potential conflict of interest.
